# Supplementary material for: Activation of JUN in fibroblasts promotes pro-fibrotic programme and modulates protective immunity
Source: Nat Commun. 2020 Jun 3;11:2795. doi: 10.1038/s41467-020-16466-4 (PMC7270081; doi:10.1038/s41467-020-16466-4)
Supplement: Supplementary file 1 — Supplementary Information [file 41467_2020_16466_MOESM1_ESM.pdf]

Supplementary data

Supplementary Figures

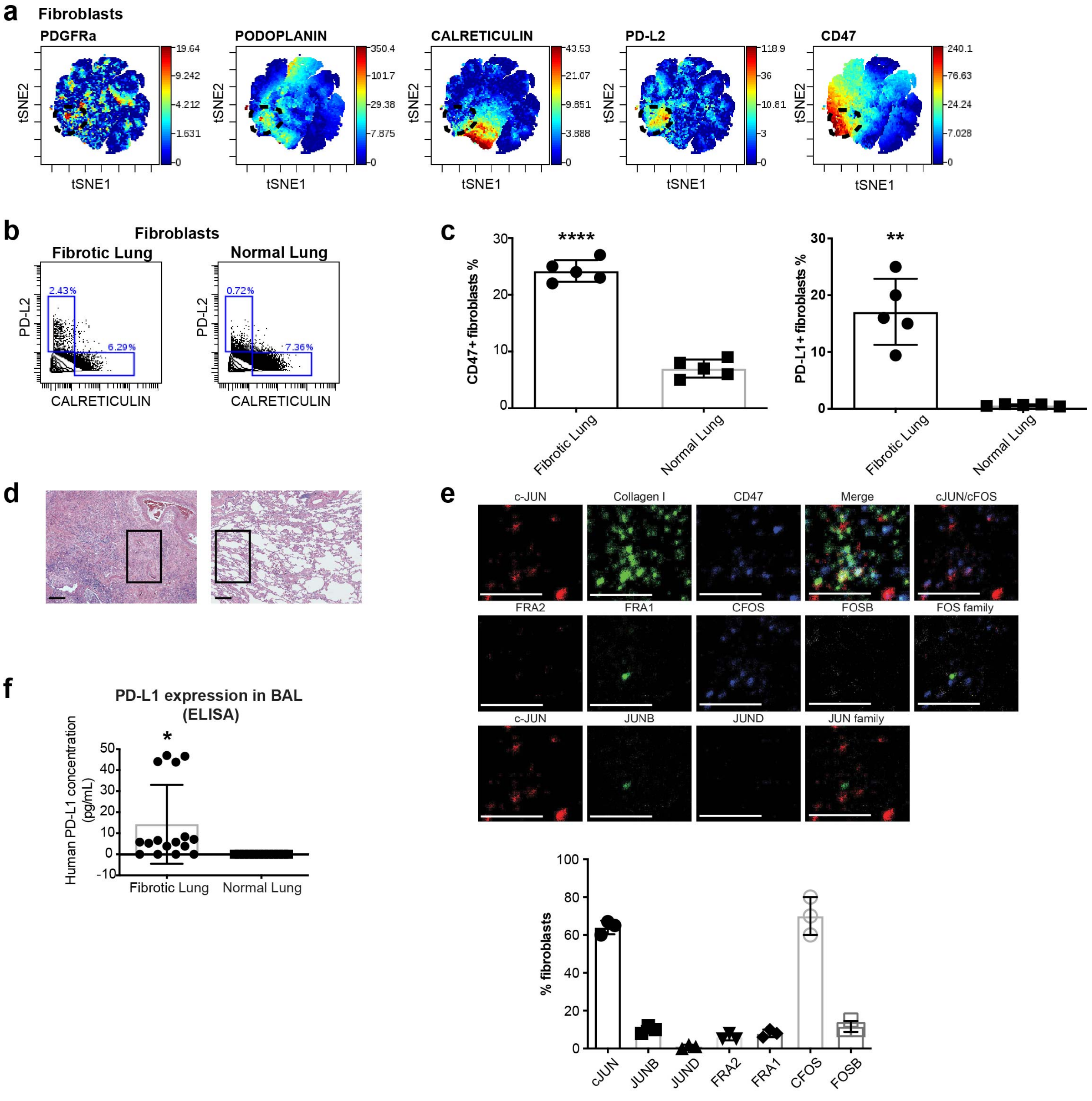

Supplementary Figure 1 (Refers to Figure 1).

(**a**) ViSNE map of concatenated fibroblasts (CD45-CD31-CK7- population) from fibrotic lung (black dotted circle) and normal lung demonstrating increased expression of PDGFRa, podoplanin, CD47 and PD-L2 but not calreticulin in subsets of fibroblasts in

fibrotic lungs. (**b**) Representative CyTOF plots of PD-L2 and calreticulin protein in fibroblasts from fibrotic and normal lungs indicating increased PD-L2 but no difference in calreticulin expression. (**c**) Quantitation of CD47 and PD-L1 immune stains in fibrotic and normal lung biopsies. Data are expressed as mean ± SD and analyzed by two-tailed unpaired *t*-test, ** $P < 0.01$; **** $P < 0.0001$. The immune stains were evaluated by a blinded pathologist, in addition to image J software. (**d**) A representative haematoxylin and eosin staining of fibrotic and normal lung tissue. The inserted black frames highlight the fibrotic and normal areas. Scale bar, 100 μm. (**e**) Multiplexed ion beam imaging (MIBI) and relevant quantitation demonstrated the co-expression of JUN and FOS with CD47 in fibroblasts in fibrotic plaques in lungs of idiopathic pulmonary fibrosis patients. Representative MIBI analysis of lung biopsy sections from 5 patients with idiopathic pulmonary fibrosis were stained with metal-conjugated antibodies. In total, 10 different markers (JUN, JUNB, JUND, FRA1, FRA2, FOS, FOSB, COLLAGEN1, CD47 and Hematoxylin) were analyzed. Eight fields of view were acquired with ten repeat scans over a single area. Experiments were run multiple times, representative examples and related analyses are shown as mean ± SD. Scale bar, 100 μm. (**f**) ELISA detected increased levels of secreted PD-L1 in fibrotic lung BAL compared to normal lungs. Data are expressed as mean ± SD of 5 fibrotic and 3 normal samples. Data were analyzed by two-tailed unpaired *t*-test, * $P < 0.05$. See **Supplementary Data 2** for statistical details.

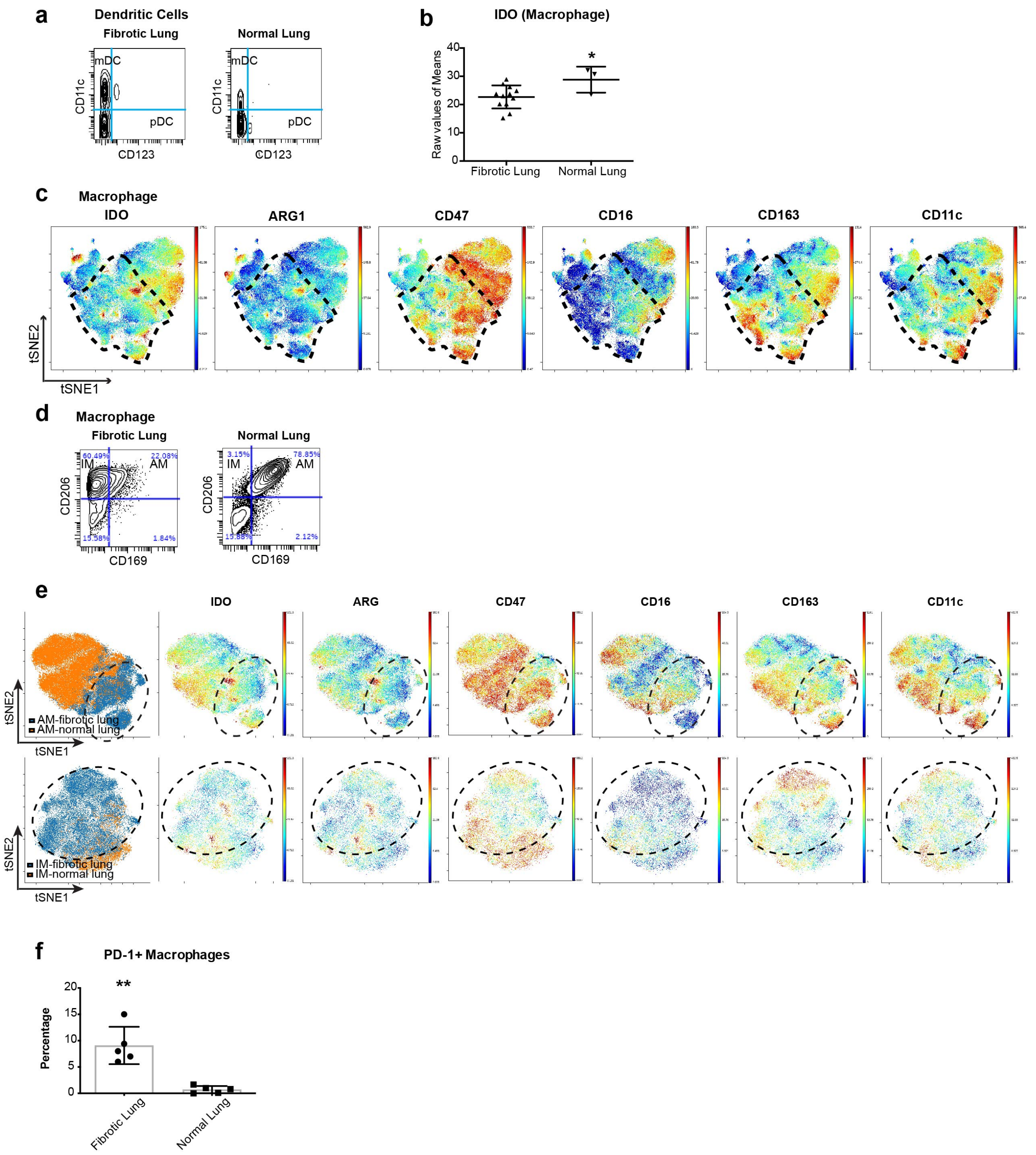

Supplementary Figure 2 (Refers to Figure 2).

(**a**) We analyzed dendritic cells in fibrotic and normal lungs with mass cytometry and found increased percentages of myeloid dendritic cells (mDC: CD45+ nonB nonT nonNK nonmacrophage CD11c+CD123-) in fibrotic lung but no difference for plasmacytoid

dendritic cells (pDC: CD45+ nonB nonT nonNK nonmacrophage CD11c-CD123+). (**b**) IDO protein expression in macrophages from fibrotic lungs is decreased compared to macrophages from normal control lungs. Raw values of means of CyTOF data are displayed on a per-patient basis with mean ± SD of 11 fibrotic and 3 normal samples and analyzed by two-tailed unpaired *t*-test, * $P < 0.05$. (**c**) The viSNE maps colored by intensity of expression (red is high, and blue is low) demonstrate the expression of IDO, ARG1, CD47, CD16, CD163 and CD11c in macrophages derived from fibrotic lungs which clustered spatially within the black circled area. (**d**) Representative histogram of mass cytometry data demonstrates decreased alveolar macrophages (AM) but increased interstitial macrophages (IM) in human fibrotic lungs. (**e**) Individual viSNE analysis of AM and IM from fibrotic lung (blue) and normal lungs (orange) suggested the immunophenotypes of AM and IM in the fibrotic tissues are clearly different from those in the normal lungs. Macrophages derived from fibrotic lungs are highlighted by the dotted black circles. (**f**) Quantitation of PD-1+ expression on macrophages (CD68+) in fibrotic and normal lung biopsies. Data are expressed as mean ± SD and analyzed by unpaired *t* test with Welch's correction (Two-tailed), ** $P < 0.01$. The immune stains were evaluated by a blinded pathologist, in addition to image J software. See **Supplementary Data 2** for statistical details.

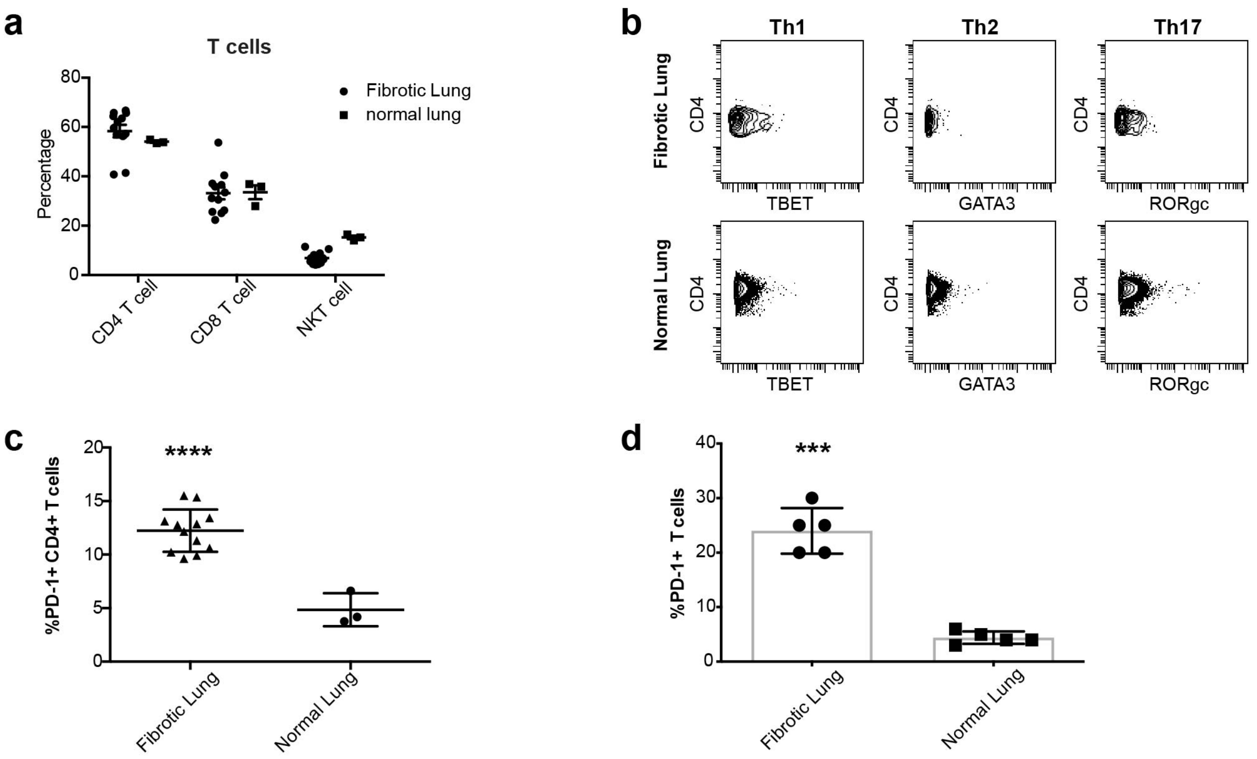

Supplementary Figure 3 (Refers to Figure 3).

(**a**) We quantified the frequencies of T cells and found no significant differences in total CD4 and CD3 T cells, but decreased percentages of NKT cells in individual samples (plotted as a fraction of total T cells and displayed as mean ± SD). (**b**) Representative plots of mass cytometry data showing no significant difference of CD4 T-cell subsets, polarized Th1 (CD3+CD4+TBET+), Th2 (CD3+CD4+GATA3) and Th17 (CD3+CD4+RORgc) in fibrotic and normal lungs. (**c**) Percentage of PD-1+ expressing CD4+ T cells are displayed with mean ± SD of 11 fibrotic and 3 normal lung samples. Data are expressed as mean ± SD and analyzed by two-way unpaired *t*-test, **** $P < 0.0001$. (**d**) Quantitation of PD-1+ expression on T cells (CD3+) in fibrotic and normal lung biopsies. Data are expressed as mean ± SD and analyzed by unpaired *t* test with Welch's correction (Two-tailed), *** $P < 0.001$. The immune stains were evaluated by a blinded

pathologist, in addition to image J software. See **Supplementary Data 2** for statistical details.

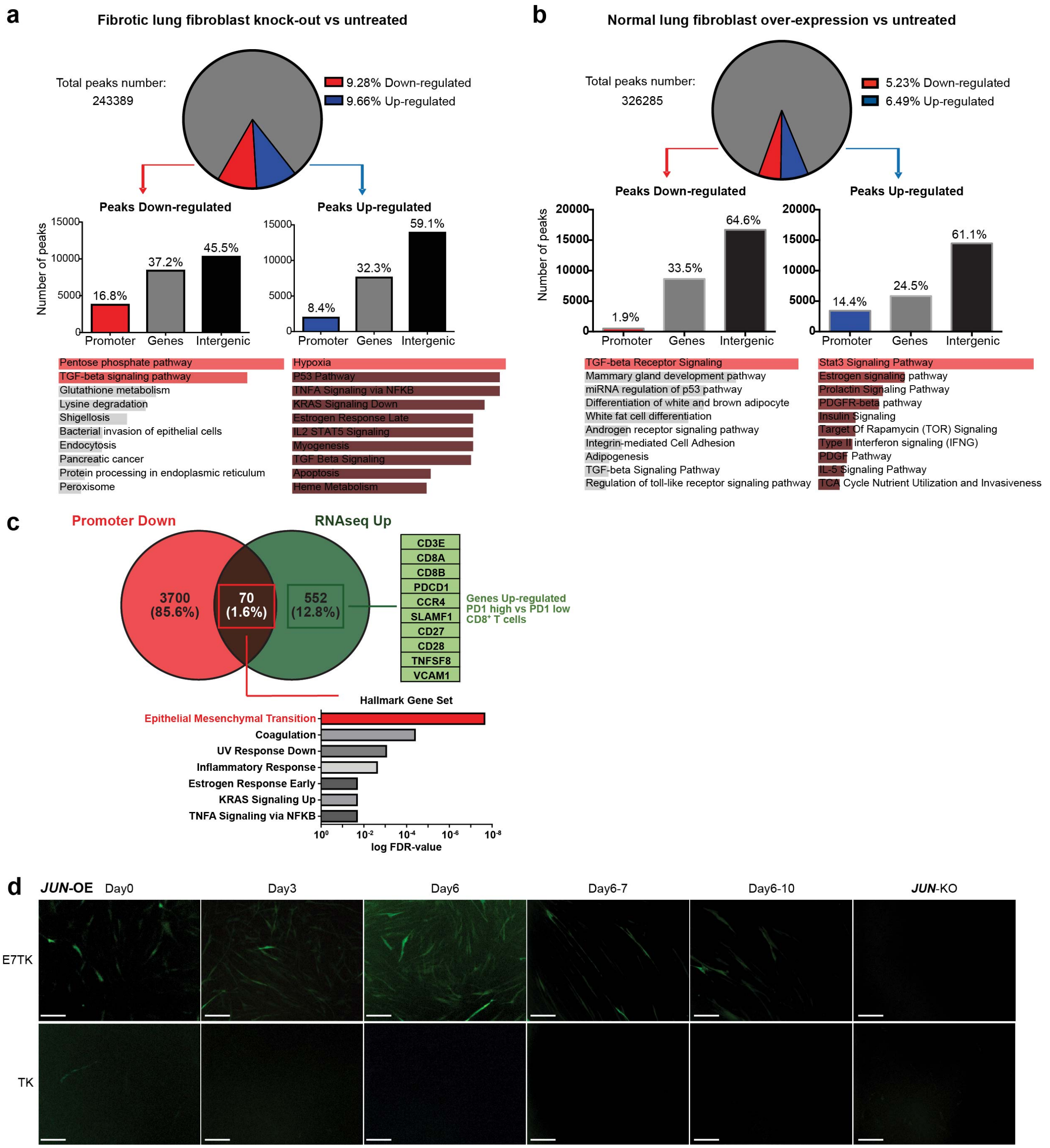

Supplementary Figure 4 (Refers to Figure 4).

(**a, b**) Quantitative comparative analysis of ATAC-seq peaks obtained from fibrotic lung fibroblasts with and without *JUN* deletion as well as normal lung fibroblasts with or without *JUN* overexpression. The top ten significant pathways which were associated with down regulation (labeled as Promoter Down in red) or up regulation (labeled as Promoter Up in blue) of the promoters were shown. (**c**) Venn Diagram generated by comparing downregulated promoters in fibrotic lung fibroblasts after *JUN* deletion with published RNA-seq data of bulk fibrotic lung samples demonstrating that 1.6% or 70 of the genes which overlapped between these two distinct data sets encoded profibrotic pathways (red) and pathways which encoded T-cell exhaustion (green). (**d**) Reporter assays for the *CD47* enhancer demonstrating continuously increasing activation of the *CD47* enhancer (E7TK) reflected by increased *EGFP* expression with increased *JUN* expression (*JUN*-OE) while the *CD47* enhancer activity decreased with doxycycline removal (turns *JUN* off) in a timely dependent manner and *JUN* deletion with CRISPR-Cas9 knock-out (*JUN*-KO) abolished the enhancer activity. Meanwhile the control TK vector showing no differences with *JUN* modification. Scale bar, 100 μm. See **Supplementary Data 2** for statistical details.

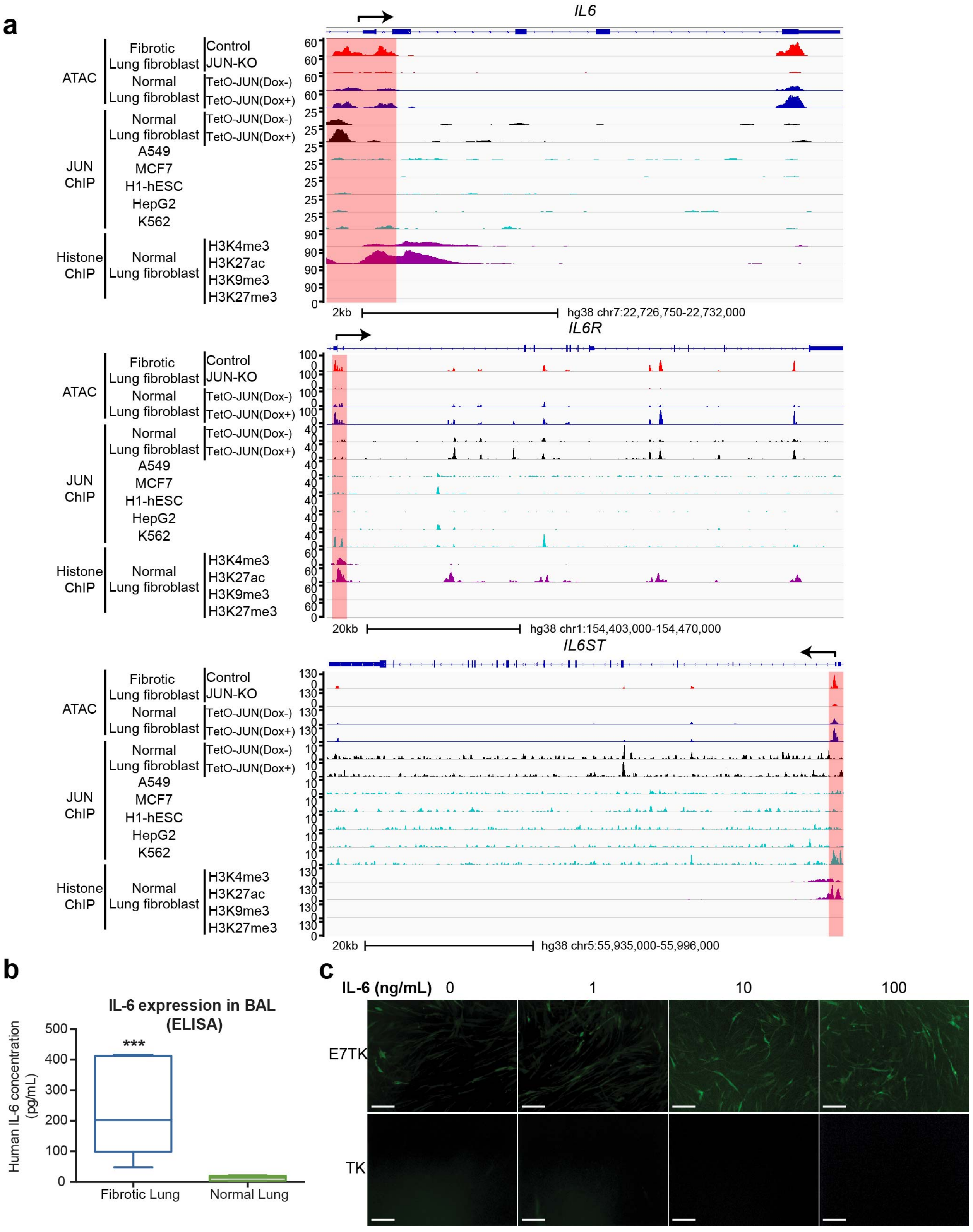

Supplementary Figure 5 (Refers to Figure 5).

(**a**) Schematic maps showing that the promoter sites (highlighted in red) for *IL-6*, *IL-6R*, and *IL-6ST* depended on *JUN* expression in normal lung fibroblasts with (TetO-*JUN* Dox+) or without (TetO-*JUN* Dox-) *JUN* overexpression and fibrotic lung fibroblasts with (*JUN*-KO) or without (Control) *JUN* knockout with CRISPR-Cas9 but not in other cell lines like A549, MCF7, h1-hESC, HepG2 and K562. We also compared our data to publicly available H3K4me3 or H3K27Ac (=histone mark for open chromatin), H3K9me3 or H3K27me3 (=histone mark for closed chromatin) ChIP-seq data generated from normal human lung fibroblast from published data to confirm the regions of open chromatin for the *IL-6* family members. (**b**) IL-6 expression in the bronchoalveolar lavages (BAL) of fibrotic and normal lungs were measured by ELISA showing dramatically increased secreted IL-6 protein. Data are expressed as min to max of 5 fibrotic and 3 normal samples. Data were analyzed by unpaired *t* test with Welch's correction (Two-tailed), *** $P < 0.001$. (**c**) *CD47* constituent enhancer-driven EGFP reporter (E7TK) expression was activated and increased in lung fibroblast cells treated with IL-6 in a dose dependent manner. Control cells were transduced with the lentiviral cassette containing the thymidine kinase (TK) minimal promoter only. Scale bar, 100 µm. See **Supplementary Data 2** for statistical details.

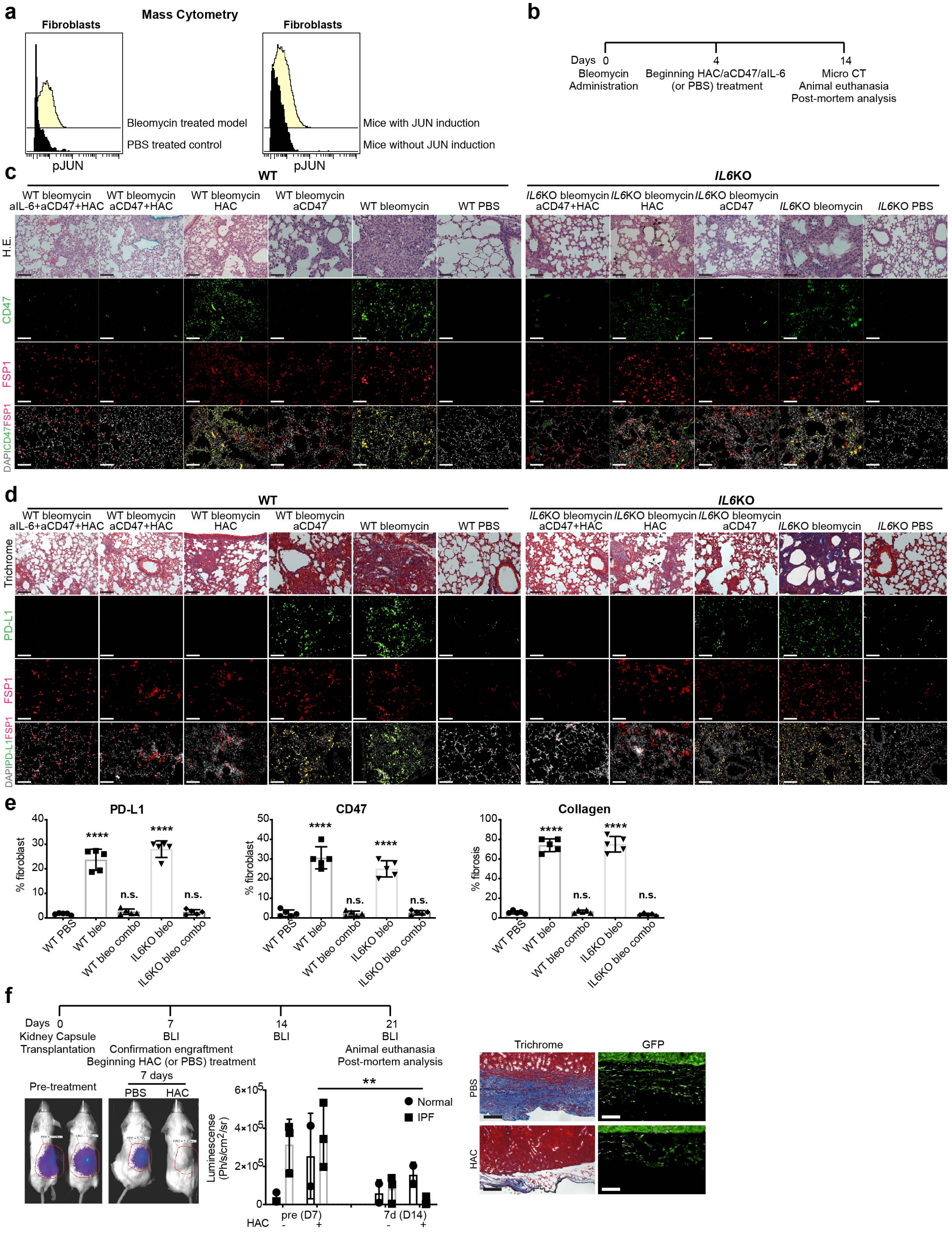

Supplementary Figure 6 (Refers to Figure 6).

(**a**) Histogram plots of mass cytometry data of phosphor p-JUN expression in lung fibroblasts comparing two different mouse models of lung fibrosis—the bleomycin-induced lung fibrosis model abundantly used by many labs and the JUN-induced lung fibrosis model—both demonstrated increased activation and phosphorylation of JUN after initiation of lung fibrosis in mice. (**b**) The time course of bleomycin induction in mice and *in vivo* treatment with blocking antibodies. (**c, d**) Morphological and molecular markers of representative histologic sections of wildtype and B6.129S2-$Il6^{tm1Kopf}$/J (*IL-6KO*) mice lung tissues after fibrosis induction and treatment with blocking antibodies against immune checkpoint inhibitors and IL-6. Hematoxylin-Eosin (H.E.) stains and CD47, FSP1 counterstained with DAPI (**c**), Masson's Trichrome stains and PD-L1 and FSP1 with DAPI (**d**) demonstrating improved fibrosis along with decreased CD47 and PD-L1 immune checkpoint protein expression in fibroblasts (FSP1+). Scale bar, 100 µm. (**e**) Quantitation of PD-L1 and CD47 expression in fibroblasts and collagen fibrosis of 10 high power fields (40x) of trichrome-stained sections. Data are expressed as mean ± SD, ordinary one-way ANOVA (Dunnett's multiple comparisons test), n.s., non-significant; **** $P < 0.0001$. The immune stains were evaluated by a blinded pathologist, in addition to image J software. (**f**) *In vivo* analysis of human fibrotic fibroblasts in kidney capsule adoptive transfer assay in NSG mice to study efficacy of PD-1/PD-L1 blockade with HAC protein. Representative bioluminescence imaging (BLI) image and quantification of luminescence intensity, trichrome and anti-GFP staining of kidney area with the xenograft demonstrate that PD-1/PD-L1 blockade with HAC increased fibrotic fibroblast clearance compared to placebo (PBS). Data are expressed as mean ± SD and analyzed by using two-way ANOVA

followed by Tukey's multiple comparisons test. ** $P < 0.01$. Scale bar, 100 μm. See **Supplementary Data 2** for statistical details.

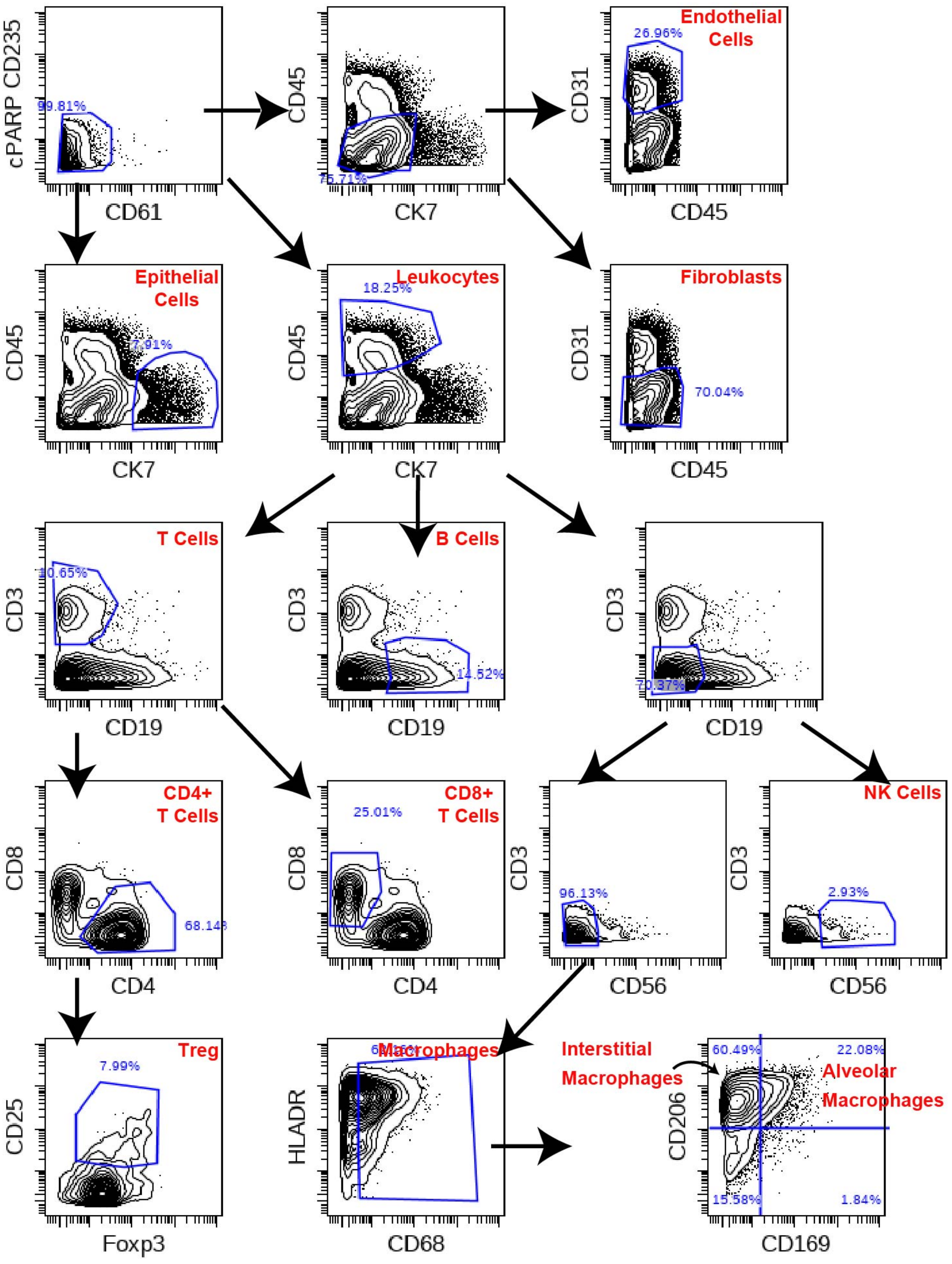

cPARP CD235
CD61
99.81%
CD45
CK7
75.71%
CD31
CD45
26.96%
Endothelial Cells
Epithelial Cells
7.91%
Leukocytes
18.25%
Fibroblasts
70.04%
T Cells
10.65%
CD3
CD19
B Cells
14.52%
70.37%
CD4+ T Cells
CD8
CD4
68.14%
CD8+ T Cells
25.01%
CD56
96.13%
NK Cells
2.93%
Treg
7.99%
CD25
Foxp3
Macrophages
HLADR
CD68
Interstitial Macrophages
Alveolar Macrophages
60.49%
22.08%
15.58%
1.84%
CD206
CD169

Supplementary Figure 7

Representative contour plots showing gating strategy for all the populations involved in this paper.

Supplymentary Table 1. Patient infomations.

Patient #	Age (years)	Gender	Ethnicity	Smoking Status	IPF Stage	Pathology	Oxygen in LPM rest	DLCO % predicted	Comorbidities
1	61	Male	Asian	Non-smoker	endstage fibrosing ILD	NSIP, PAH, BAC, honeycomb	6-8	20	GERD
2	69	Male	Caucasian	Former smoker-	endstage fibrosing ILD	UIP, honeycomb	6	22	GERD, DM type II
3	72	Male	Caucasian	Former smoker- quit 1976	endstage fibrosing ILD	UIP, honeycomb	4-6	<30	none
4	65	Female	Caucasian	2nd had exposure, Non-smoker	endstage fibrosing ILD	UIP, honeycomb,	4-6	25%	Hypercholesterolemia, DM type II
5	69	Male	Caucasian	Former smoker	endstage fibrosing ILD	UIP, PAH, honeycomb	8	<20	ILD, GERD, depression
6	72	Male	Caucasian	Never smoker	endstage fibrosing ILD	UIP, honeycomb	8	22	GERD, HTN, Sleep apnea, ILD, skin cancer, BCC, SCC
7	68	Male	Caucasian	Former smoker- quit 1980	endstage fibrosing ILD	NSIP	6	20	SSC, psoriasis, OSA, HTN, obese
8	68	Male	Caucasian	Former smoker- quit 1990, 25 pack years	endstage fibrosing ILD	UIP, honeycomb	6	20	GERD, HTN, CKD
9	63	Male	Caucasian	Former smoker- quit 1995, 25 pack years	endstage fibrosing ILD	UIP, honeycomb	10	18	MCI, CAD, HTN, DM type II, hyperlipid, GERD, depression
10	54	Male	Hispanic	Former smoker	endstage fibrosing ILD	chronic hypersens pneumonitis,	4	19	ILD, GERD, DM type II, TBC
11	50	Female	Asian	Non-smoker	endstage fibrosing ILD	chronic hypersens pneumonitis, PAH	4	23	GERD, HTN, hyperlipid, DM type II
12	59	Male	Caucasian	Former smoker	normal lung tissue, lobectomy#	normal lung tumor: lung adenoCA	N/A	N/A	lung adenoCA
13	66	Male	Caucasian	Former smoker	normal lung tissue, lobectomy#	normal lung tumor: lung adenoCA	N/A	N/A	lung adenoCA
14	72	Male	Caucasian	Former smoker	normal lung tissue, lobectomy#	normal lung tumor: lung adenoCA	N/A	N/A	lung adenoCA

*All the ILD patients included in the studies had histologic or radiographic evidence of end-stage fibrosing interstitial lung disease (ILD): UIP (8), fibrotic NSIP (2), fibrotic chronic interstitial pneumonitis (2), DLCO all severe decreased DLCO between <25% of predicted ,FVC <80%, FVC 10% or greater decrement in FVC during 6-month follow-up, 6 minute walk pulse oximetry below 88% or 50m decline in over 6 months, patients **associated pulmonary hypertensive (PAH) features on histopathology. # Our healthy control lung specimens were derived from lung lobectomy specimens for lung cancer, we only received histologic healthy appearing lung distant from the tumor (lung specimen weights 150-200g, tumor diameters ranging 0.8-2 cm, stage pT2pN0).

Supplymentary Table 2. Cell counts.

Patient ID	Total live	Fibroblasts	Macrophage
PF-1	54734	26626	5209
PF-2	204489	82094	11889
PF-3	210786	19595	15715
PF-4	16770	5104	1903
PF-5	27951	1969	9563
PF-6	79863	7778	17634
PF-7	198291	149534	7322
PF-8	21353	10575	2270
PF-9	86505	34367	17169
PF-10	50439	31280	971
PF-11	52805	11608	2409
NC-1	175103	147576	2821
NC-2	180465	58518	27427
NC-3	215871	34424	34238

Supplymentary Table 3. Term defination.

PF	pulmonary fibrosis
Epi	epithelial cells
NK	natural killer cells
pDC	plasmacytoid dendritic cells
EC	endothelial cells
MAC	macrophages
ATAC-seq	Assay for Transposase Accessible Chromatin using sequencing
ChIP-seq	chromatin immunoprecipitation sequencing
CYTOF	mass cytometry
PCA	Principal component analysis
BAL	bronchoalveolar lavage
MIBI	multiplexed ion beam imaging
TAM	Tumor-associated macrophages
NSG	NOD.Cg-PrkdcscidIl2rgtm1Wjl/SzJ, NOD scid gamma
PD-L1	CD274
SIRPa	CD172a
IL-6	interleukin 6
DC	dendritic cells
AM	alveolar macrophages
IM	interstitial macrophages
PBMC	peripheral blood mononuclear cell
SMA	smooth muscle actin
HAC	high-affinity consensus (HAC) PD-1, an engineer nonantibody biologic based on the ectodomain of PD-1 which was more effective than anti–PD-L1 antibodies
IDO	Indoleamine 2,3-dioxygenase
KO	knock out
OE	over expression
WT	wild type
Dox	doxycycline
Th	T helper
IL-6KO mice	B6.129S2-*Il6*^{tm1Kopf}/J mice
TK	thymidine kinase
E7	CD47 functional enhancer E7

Supplymentary Table 4. Taqman probe.

JUN	Hs01103582_s1
CD47	Hs00179953_m1
CD274	Hs00204257_m1
IL-6	Hs00174131_m1
