# Supplementary material for: Activation of JUN in fibroblasts promotes pro-fibrotic programme and modulates protective immunity
Source: Nat Commun. 2020 Jun 3;11:2795. doi: 10.1038/s41467-020-16466-4 (PMC7270081; doi:10.1038/s41467-020-16466-4)
Supplement: Supplementary file 3 — Description of Additional Supplementary Files [file 41467_2020_16466_MOESM3_ESM.docx]

**Description of Additional Supplementary Files**

**File Name: Supplementary Data 1**

**Description:** Antibody informations

**File Name: Supplementary Data 2**

**Description:** Statistical information
