# Supplementary material for: Activation of JUN in fibroblasts promotes pro-fibrotic programme and modulates protective immunity
Source: Nat Commun. 2020 Jun 3;11:2795. doi: 10.1038/s41467-020-16466-4 (PMC7270081; doi:10.1038/s41467-020-16466-4)
Supplement: Supplementary file 6 — Reporting Summary [file 41467_2020_16466_MOESM6_ESM.pdf]

Corresponding author(s): Gerlinde Wernig

Last updated by author(s): Dec 10, 2019

Reporting Summary

Nature Research wishes to improve the reproducibility of the work that we publish. This form provides structure for consistency and transparency in reporting. For further information on Nature Research policies, see Authors & Referees and the Editorial Policy Checklist .

Statistics

For all statistical analyses, confirm that the following items are present in the figure legend, table legend, main text, or Methods section.

n/a	Confirmed	
☐	☒	The exact sample size (*n*) for each experimental group/condition, given as a discrete number and unit of measurement
☐	☒	A statement on whether measurements were taken from distinct samples or whether the same sample was measured repeatedly
☐	☒	The statistical test(s) used AND whether they are one- or two-sided<br>*Only common tests should be described solely by name; describe more complex techniques in the Methods section.*
☒	☐	A description of all covariates tested
☒	☐	A description of any assumptions or corrections, such as tests of normality and adjustment for multiple comparisons
☐	☒	A full description of the statistical parameters including central tendency (e.g. means) or other basic estimates (e.g. regression coefficient) AND variation (e.g. standard deviation) or associated estimates of uncertainty (e.g. confidence intervals)
☐	☒	For null hypothesis testing, the test statistic (e.g. *F*, *t*, *r*) with confidence intervals, effect sizes, degrees of freedom and *P* value noted<br>*Give P values as exact values whenever suitable.*
☒	☐	For Bayesian analysis, information on the choice of priors and Markov chain Monte Carlo settings
☒	☐	For hierarchical and complex designs, identification of the appropriate level for tests and full reporting of outcomes
☒	☐	Estimates of effect sizes (e.g. Cohen's *d*, Pearson's *r*), indicating how they were calculated

Our web collection on statistics for biologists contains articles on many of the points above.

Software and code

Policy information about availability of computer code

Data collection	Single Cell Mass Cytometry was measured on a CyTOF mass cytometer (Fluidigm). CT Scans were performed using a Bruker Skycan 1276 (Bruker, Belgium). ATAC and ChIP-sequence library was sequenced using 50-bp × 2 paired-end reads on an Illumina HiSeq 2500 system. CT scans were then analyzed with the Bruker Skycan tools. Luciferase-based optimal imaging was performed using a Lago optical imaging system (Spectral imaging instruments, AZ, USA). Analysis was done with the Aura Software from the same manufacturer.
Data analysis	microscope images were analyzed with ImageJ (v1.52, NIH). ViSNE maps were generated with software tools available at https://www.cytobank. org/ by considering all surface markers. ATAC-seq and ChIP-seq data analysis used the following tools and versions: Cutadapt v1.9.1, Picard v1.126, Bowtie2 v2.2.8, MACS2 v2.1.0.20150731, and Bedtools v 2.26. Statistical analyses were performed using Prism software (GraphPad Software).

For manuscripts utilizing custom algorithms or software that are central to the research but not yet described in published literature, software must be made available to editors/reviewers. We strongly encourage code deposition in a community repository (e.g. GitHub). See the Nature Research guidelines for submitting code & software for further information.

Data

Policy information about availability of data

All manuscripts must include a data availability statement. This statement should provide the following information, where applicable:

- Accession codes, unique identifiers, or web links for publicly available datasets
- A list of figures that have associated raw data
- A description of any restrictions on data availability

Raw ATAC-seq and ChIP-seq data have been deposited in the Gene Expression Omnibus (GEO) database under accession code GSE114844. JUN ChIP-seq of HepG2 (GSM935364), MCF-7 (GSE91550), H1-hESC (GSM935614), A549 (GSE92221) and K562 (GSM1003609), Histone ChIP-seq data of H3K4me3 (GSM733723), H3K27ac (GSM733646), H3K9me3 (GSM1003531) and H3K27me3 (GSM733764) and RNA-seq data of fibrotic lungs (GSE52463) are from the public GEO database.

Field-specific reporting

Please select the one below that is the best fit for your research. If you are not sure, read the appropriate sections before making your selection.

☒ Life sciences ☐ Behavioural & social sciences ☐ Ecological, evolutionary & environmental sciences

For a reference copy of the document with all sections, see nature.com/documents/nr-reporting-summary-flat.pdf

Life sciences study design

All studies must disclose on these points even when the disclosure is negative.

Sample size	Experimental conditions (number of independent experiments, numbers of cells or fields per experiment...) were chosen according to standard procedures in cell biology research, and statistical significance was tested on the corresponding results.
Data exclusions	N/A
Replication	All replicates reported in the manuscript.
Randomization	Samples were not randomized for the experiments. No randomization of mice. Mice analysis were litter mates and sex-matched whenever possible.
Blinding	Investigators were not blinded to cell/mouse genotypes during experiments.

Reporting for specific materials, systems and methods

We require information from authors about some types of materials, experimental systems and methods used in many studies. Here, indicate whether each material, system or method listed is relevant to your study. If you are not sure if a list item applies to your research, read the appropriate section before selecting a response.

Materials & experimental systems

n/a	Involved in the study	
☐	☒	Antibodies
☒	☐	Eukaryotic cell lines
☒	☐	Palaeontology
☐	☒	Animals and other organisms
☐	☒	Human research participants
☒	☐	Clinical data

Methods

n/a	Involved in the study	
☐	☒	ChIP-seq
☐	☒	Flow cytometry
☒	☐	MRI-based neuroimaging

Antibodies

Antibodies used	All the antibody informations were provided in the supplemental tables.
Validation	Reported from the companies that provide the antibodies.

Animals and other organisms

Policy information about studies involving animals; ARRIVE guidelines recommended for reporting animal research

Laboratory animals	C57BL/6 and B6.129S2-Il6 tm1Kopf/J mice, 8-10 weeks of age were purchased from Jackson laboratory.
Wild animals	N/A
Field-collected samples	N/A
Ethics oversight	Mice were maintained in Stanford University Laboratory Animal Facility in accordance with Stanford Animal Care and Use Committee and National Institutes of Health guidelines (SU-APLAC 30912).

Note that full information on the approval of the study protocol must also be provided in the manuscript.

Human research participants

Policy information about studies involving human research participants

Population characteristics	All the related info about population charateristics has been provided in the Supplementary table 1
Recruitment	We received primary lung tissues exclusively from patients with end-stage pulmonary fibrosis undergoing transplantation. Therefore, our cohort of patients represents severely fibrotic lung disease. It has been challenging to receive normal control lung tissues. While we have received lung tissues from normal lung resections from tumor resections from Stanford tissue bank as well as lungs from rapid autopsies, it appeared that the only normal lung tissues harvested during surgery by the tissue bank were of sufficient viability to include in our CyTOF studies; while other cell-type fractions appeared representative we noted a bias towards less endothelial cells in the normal biopsies due to the relatively small amounts of lung tissue we received from the tissue bank.
Ethics oversight	Human samples were obtained under Stanford University's IRB approval.

Note that full information on the approval of the study protocol must also be provided in the manuscript.

ChIP-seq

Data deposition

☒ Confirm that both raw and final processed data have been deposited in a public database such as GEO.

☒ Confirm that you have deposited or provided access to graph files (e.g. BED files) for the called peaks.

Data access links<br>*May remain private before publication.*	https://www.ncbi.nlm.nih.gov/geo/query/acc.cgi?acc=GSE114844
Files in database submission	ChIP_NLF_R1_001.fastq fastq<br>ChIP_NLF_R2_001.fastq fastq<br>ChIP_NLF_OE_R1_001.fastq fastq<br>ChIP_NLF_OE_R2_001.fastq fastq<br>ChIP_input_NLF_R1_001.fastq fastq<br>ChIP_input_NLF_R2_001.fastq fastq<br>ChIP_input_NLF_OE_R1_001.fastq fastq<br>ChIP_input_NLF_OE_R2_001.fastq fastq<br>ChIP_NLF.regionPeak regionPeak<br>ChIP_NLF_OE.regionPeak regionPeak<br>ChIP_NLF.signal.bigwig bigwig<br>ChIP_NLF_OE.signal.bigwig bigwig
Genome browser session<br>(e.g. UCSC)	no longer applicable

Methodology

Replicates	All bioChIP-seq experiments were done in paired replicates.
Sequencing depth	Sample rep. Distinct Reads (Pairs) total reads length sequence type<br>ChIP_NLF_R1_001 2 22215625 79305622 75 Paired-end<br>ChIP_NLF_INPUT_R1_001 2 15692059 140558580 75 Paired-end<br>ChIP_NLF_OE_R1_001 2 9286555 73581338 75 Paired-end<br>ChIP_NLF_OE_INPUT_R1_001 2 10528100 73590810 75 Paired-end
Antibodies	The antibody for ChIP-seq is c-Jun (60A8) Rabbit mAb #9165 from Cell signaling Technology.
Peak calling parameters	BioChIP-Seq: macs2 callpeak -t chip.bam -c input.bam -f BAM -g hg -n chip -p 0.01 --verbose=0
Data quality	BioChIP-seq peak numbers are listed below. Reproducible peaks were identified using IDR (ENCODE metrics) at IDR_THRESHOLD=0.05 between each set of replicate data files. Each IDR peak was represented by the MACS2 summit ± 100 bp of the individual replicate with the greatest peak intensity.<br>Sample rep. overlap peak number<br>ChIP_NLF_R1_001 2 46536<br>ChIP_NLF_INPUT_R1_001 2<br>ChIP_NLF_OE_R1_001 2 33350<br>ChIP_NLF_OE_INPUT_R1_001 2
Software	ChIP-seq data analysis used the Kundaje lab pipeline. Following tools and versions: Cutadapt v 1.9.1, Picard v1.126, Bowtie2 v2.2.8, MACS2 v2.1.0.20150731, and Bedtools v 2.26. First, Nextera adaptor sequences were trimmed from the reads by using cutadapt program v 1.9.1. These reads were aligned to human genome hg38 using bowtie2. The standard default

settings were modified to allow mapped paired-end fragments up to 2 kb. Only the reads with mapping quality greater than 30 were kept, and the duplicated reads were removed using Picard tools v1.126. The reads from mitochondria were also removed, then convert PE BAM to tagAlign (BED 3+3 format) using Bedtools v 2.26 functions. Differential expression analysis were done by DESeq2 (http://bioconductor.org/packages/release/bioc/vignettes/DESeq2/inst/doc/DESeq2.html). Differential peaks with p-val less than 0.01 and absolute log2 fold change above 1.

Flow Cytometry

Plots

Confirm that:

- [x] The axis labels state the marker and fluorochrome used (e.g. CD4-FITC).
- [x] The axis scales are clearly visible. Include numbers along axes only for bottom left plot of group (a 'group' is an analysis of identical markers).
- [x] All plots are contour plots with outliers or pseudocolor plots.
- [x] A numerical value for number of cells or percentage (with statistics) is provided.

Methodology

Sample preparation	Sample preparations were described in Methods.
Instrument	Aria Iiu SORP
Software	Data were collected by BD FACSDiva, and analyzed by CYTOBANK
Cell population abundance	Not applicable
Gating strategy	FSC/SSC gating was used to exclude dead cells and debris. Doublets were excluded by plotting PI-width and PI-area.

- [x] Tick this box to confirm that a figure exemplifying the gating strategy is provided in the Supplementary Information.
